# Patient-Derived Gastric Carcinoma Xenograft Mouse Models Faithfully Represent Human Tumor Molecular Diversity

**DOI:** 10.1371/journal.pone.0134493

**Published:** 2015-07-28

**Authors:** Tianwei Zhang, Lin Zhang, Shuqiong Fan, Meizhuo Zhang, Haihua Fu, Yuanjie Liu, Xiaolu Yin, Hao Chen, Liang Xie, Jingchuan Zhang, Paul R. Gavine, Yi Gu, Xingzhi Ni, Xinying Su

**Affiliations:** 1 Asia & Emerging Markets iMed, AstraZeneca R&D, Shanghai, P.R. China; 2 Research and Development Information, AstraZeneca R&D, Shanghai, P.R. China; 3 Department of General Surgery, Ren Ji Hospital, School of Medicine, Shanghai Jiao Tong University, Shanghai, P.R. China; University of Quebec at Trois-Rivieres, CANADA

## Abstract

Patient-derived cancer xenografts (PDCX) generally represent more reliable models of human disease in which to evaluate a potential drugs preclinical efficacy. However to date, only a few patient-derived gastric cancer xenograft (PDGCX) models have been reported. In this study, we aimed to establish additional PDGCX models and to evaluate whether these models accurately reflected the histological and genetic diversities of the corresponding patient tumors. By engrafting fresh patient gastric cancer (GC) tissues into immune-compromised mice (SCID and/or nude mice), thirty two PDGCX models were established. Histological features were assessed by a qualified pathologist based on H&E staining. Genomic comparison was performed for several biomarkers including ERBB1, ERBB2, ERBB3, FGFR2, MET and PTEN. These biomarkers were profiled to assess gene copy number by fluorescent *in situ* hybridization (FISH) and/or protein expression by immunohistochemistry (IHC). All 32 PDGCX models retained the histological features of the corresponding human tumors. Furthermore, among the 32 models, 78% (25/32) highly expressed ERBB1 (EGFR), 22% (7/32) were ERBB2 (HER2) positive, 78% (25/32) showed ERBB3 (HER3) high expression, 66% (21/32) lost PTEN expression, 3% (1/32) harbored *FGFR2* amplification, 41% (13/32) were positive for MET expression and 16% (5/32) were *MET* gene amplified. Between the PDGCX models and their parental tumors, a high degree of similarity was observed for *FGFR2* and *MET* gene amplification, and also for ERBB2 status (agreement rate = 94~100%; kappa value = 0.81~1). Protein expression of PTEN and MET also showed moderate agreement (agreement rate = 78%; kappa value = 0.46~0.56), while ERBB1 and ERBB3 expression showed slight agreement (agreement rate = 59~75%; kappa value = 0.18~0.19). ERBB2 positivity, *FGFR2* or *MET* gene amplification was all maintained until passage 12 in mice. The stability of the molecular profiles observed across subsequent passages within the individual models provides confidence in the utility and translational significance of these models for *in vivo* testing of personalized therapies.

## Introduction

Gastric carcinoma (GC) is the third most common cancer worldwide and is the most frequent cancer diagnosed in East Asian countries [1]. Despite recent progress in earlier diagnosis and improved therapeutic regimens, many patients still eventually develop advanced disease and have poor clinical outcomes. The median overall survival is 8–10 months and 5-year survival is less than 7% for metastatic GC [2]. With regard to standard chemotherapy, limited efficacy has spurred research into targeted therapies designed to block signaling via molecular pathways known to be important for gastric tumorigenesis [3, 4]. To date, two monoclonal antibodies have been approved by the FDA for the treatment of GC, namely Trastuzumab [5] and Ramucirumab [6, 7], targeting ERBB2 (HER2) and VEGFR2 respectively. A number of other targeted therapeutics are currently being tested in mid to late stage GC trials including: AZD4547, targeting the *FGFR2* gene [8, 9], and Onartuzumab [10], ARQ197 [11], AMG102 [12, 13] and crizotinib [14] all targeting the MET pathway [15]. Within the drug development process, evaluation of preclinical efficacy with relevant *in vivo* models is an important checkpoint before moving the drug forward into human clinical studies. Accordingly, one of our research goals is to establish appropriate preclinical models which as accurately as possible represent the complexity of human GC and provide predictive power.

In contrast to standard cancer cell line derived xenografts, which may undergo genetic modification as well as subpopulation rearrangements during the cell line’s *in vitro* culture [16], patient-derived cancer xenograft (PDCX) models are established by directly engrafting surgically resected human tumor tissues into immune deficient mice. Therefore, at least initially, PDCX models inherit the complexity and genetic diversity of the original human tumors [17, 18] and are preferred models for evaluating the anticancer efficacy of targeted therapies [19, 20]. Panels of tumor-specific PDCX models have been established in many cancer types [21] including breast cancer [22], ovarian cancer [23], esophageal carcinoma [17], non small cell lung cancer [18, 24, 25], colorectal cancer [26–28], prostate cancer [29, 30] and pancreatic cancer [31]. The generation of patient-derived gastric cancer xenograft models (PDGCX) has been reported recently in great depth by Zhu and colleagues using gastroscopic biopsy samples from non-resectable advanced GC [32]. However, using surgical GC samples to establish PDGCX models has been more challenging. To our knowledge, not many PDGCX models have been established [33–36] apart from those established by our group [9, 37–40]. In addition, molecular biomarkers have not been well studied.

In the present study, we successfully established 32 PDGCX models from human GC surgical samples and performed histological examination and profiling of genetic biomarkers. These genetic biomarkers, which included *ERBB1 (EGFR)*, *ERBB2 (HER2)*, *ERBB3 (HER3)*, *PTEN*, *FGFR2* and *MET*, are six genes that are known targets for clinical or pre-clinical targeted therapies in GC. Through comparison to parental patient tumors, we demonstrated that these PDGCX models accurately maintained the histological and genetic characteristics of human GC, thereby underscoring their value and potential predictive power in evaluating oncology drug efficacy in pre-clinical studies.

## Material and Methods

### Patients and tumor samples

GC tissues from 207 treatment-naïve patients were obtained intraoperatively during gastrectomy resection at Ren Ji Hospital (Shanghai, China) from 2009 to 2012. Prior written informed consent was obtained from all patients and the study protocol was approved by the ethics committee at Ren Ji hospital. Resected tumor samples were separated into two parts. One part was used for *in vivo* engrafting as described in the next paragraph, while another part was processed to generate formalin-fixed, paraffin-embedded (FFPE) tissues blocks. FFPE sections were stained with hematoxylin and eosin (H&E) and reviewed by pathologist to confirm the GC diagnosis.

### Establishment of PDGCX models

All animal experiments were performed in accordance of the guidelines (IACUC protocol NO.1404-ONM-01) approved by AstraZeneca Institutional Animal Care and Use Committee (IACUC). PDGCX mouse models were established using fresh GC tissues surgically removed from GC patients. In brief, surgically removed GC tissues (F0) were immediately placed into FBS-free medium with 50units/ml penicillin and 50ug/ml streptomycin, and then transported to the animal facility within two hours for implantation into immune compromised mice. The tissues were cut into 2 mm^3^ fragments and subsequently transplanted subcutaneously into the right hind flanks of immune-compromised mice, either 8-10-week-old nude (nu/nu) or SCID mice (Vital River, Beijing, China). In general, tissue from one patient tumor was used to implant into 5~10 mice. Specific standard operation procedure (SOP) and policy have been generated for alleviation of pain, distress or discomfort for routine monitor, care and humanized euthanasia. The criteria for humane euthanasia in mouse experiments, which has been defined in the IACUC protocol and SOP, include animals in a moribund state; ≥20% body weight loss which can’t be recovered in 5 days; tumor mass **≥** 2cm in any dimension or volume over 2cm^3^ and ulcerated or necrotic masses of engraft, etc. If any clinical signs in implanted animals were found to meet the criteria for humanized euthanasia, euthanasia was performed by excessive CO_2_ inhalation. Subcutaneous tumor growth in mice was observed daily until 90 days. Xenograft tumors were measured once a week using calipers when tumors started to grow up in mice. Tumor volume was calculated using the formula V = π/6 × length × width × width, with the length and width value being obtained from caliper measurements. When the first generation (F1) of the xenografted tumors reached a size of 1–2 cm^3^, tumor-bearing mice were sacrificed and the xenograft tumor tissues were resected under aseptic conditions and implanted into nude mice for maintenance and expansion (F2) within 30 minutes of resection. Meanwhile, representative portions of freshly harvested tissues were fixed in 10% formalin buffer for 24 hours and embedded in paraffin (FFPE) for pathological assessment. A PDGCX model was considered successfully established if it was passagable in nude mice for more than three passages (F3). Xenograft tumor tissues from well-established PDGCX models were also harvested every passage for model characterization and biomarker studies.

### Immunohistochemistry (IHC)

IHC assays were performed to detect the protein expression of 5 biomarkers. All IHC assays were done on 4um FFPE tissue slides (SuperFrost, ThermoFisher), sectioned from FFPE blocks 24 hours prior to experiment. For ERBB2 staining, a HercepTest kit (K5204, DAKO) was used following the manufacturer’s instructions. MET staining was performed using a rabbit monoclonal anti-total cMET (SP44) antibody (790–4430, Ventana Medical Systems) on a Ventana automatic immunostainer (Discovery XT; Ventana Medical Systems). ERBB1, ERBB3 and PTEN staining were performed manually using the following antibodies: rabbit anti-tERBB1 monoclonal antibody (1:100 dilution, 0.00017ug/ul, CST4267, Cell Signaling Technology), anti-ERBB3 antibody (1:2000 dilution, CST D43D4, 0.0009ug/ul, Cell Signaling Technology) and rabbit anti-PTEN monoclonal antibody (1:100 dilution, 0.00026ug/ul, CST9559, Cell Signaling Technology). The manual IHC procedure is briefly described as follows: slides were dried at 37°C overnight and then baked at 56°C for 30min, then deparaffinized in xylene and rehydrated through a graded series of ethanol concentrations. Antigen retrieval was performed in a pressure cooker using Target Retrieval Solution, pH 9 (K8004, DAKO) for ERBB1 and ERBB3 staining, or pH6 (K8005, DAKO) for PTEN staining for 5 min. Intrinsic peroxidase activity was blocked by peroxidase blocking solution (S2023, DAKO) for 5 min. Slides were then covered with primary antibody solution and incubated at room temperature for 60min. After two washes in TBS-T for 5 min each, slides were incubated with visualization reagent at room temperature for 30 min. Following two additional washes in TBS-T, slides were visualized using DAB substrate-chromagen (K3468, DAKO). Sections were then dehydrated through a graded series of ethanol concentrations, cleared in xylene and coverslipped in DPX mounting medium. Images were taken by Photoshop CS3 using Leica DM2500 microscope under 40X objective.

### Fluorescence *in situ* hybridization (FISH)

Dual-color FISH was performed to assess copy number changes in three oncogenes including *ERBB2*, *FGFR2* and *MET*. The *ERBB2*/CEP17 dual color FISH probe (30–171060, Vysis) was purchased from Vysis Company, the *FGFR2* and *MET* FISH probes were generated internally by directly labeling BAC (*FGFR2*: RP11-62L18; *MET*: CTD-2270N20) DNA with Red-dUTP (02N34-050, ENZO). The CEP10- Spectrum Green probe (Vysis, Cat # 32–132010) and CEP7- Spectrum Green probe (32–132007, Vysis) for the centromeric regions of chromosome 10 and chromosome 7 were used as internal controls for the *FGFR2* and *MET* probes respectively. Detailed procedures were previously described [41]. In brief, assays were run on 4 micron dewaxed and dehydrated FFPE samples, or 3 micron snap-frozen tissues. The SpotLight Tissue pretreatment Kit (00–8401, Invitrogen) was used for pretreatment according to the manufacturer’s instructions. Sections and probes were codenaturated at 79°C for 6 minutes and then hybridized at 37°C for 48 hours (FFPE) or 24 hours (frozen tissues). After a quick post wash off process (0.3%NP40/2xSSC at 75.5°C for 2 minutes, twice in 2×SSC at room temperature for 2 minutes), sections were mounted with 0.3μg/ml DAPI (H-1200, Vector).

FISH signals were observed using a fluorescence microscope equipped with the appropriate filters to allow visualization of the intense red/green signals and the blue counterstained nuclei. For *ERBB2* analysis, tumors with a ratio of *ERBB2* to CEP17 ≥2 were defined as amplified; for *FGFR2* analysis, tumors with a ratio of *FGFR2* to CEP10 ≥2 were defined as amplified and tumors with an average *MET* gene copy number of greater than 5 was defined as amplified.

### Criteria for defining biomarker positivity

Tumors were categorized as ERBB1/ERBB3/MET expression positive when the IHC staining signal intensity was 2+ or 3+ (0–3+ scale) in >10% of tumor cells. For PTEN, tumors with an IHC staining signal intensity of 0 (0–3+ scale) were categorized as ‘PTEN loss’. *FGFR2* and *MET* FISH positivity was defined by gene amplification. ERBB2 positive cases were defined as IHC 3+ or IHC 2+ plus FISH amplification following the criteria used in the gastric TOGA trial [42].

### Statistical analysis

The analysis was conducted using R version 3.0.2. The demographic and clinical characteristics of the two groups were compared using the Fisher’s Exact Test for categorical variables or Mann-Whitney U Test for continuous variables. The difference in survival distributions between the two groups were compared using the log-rank test. Two-sided P values <0.05 were considered statistically significant. Biomarker concordance between the primary samples and the xenograft models were assessed using the Cohen’s kappa coefficient. For the extent of agreement of the estimated kappa, the judgment given by Landis J.R. and Koch G.G. was used [43].

## Results

### PDGCX model establishment

Among 207 human GC tissue engrafted mouse models, patient tumors grew up in 49 (23.7%) immunodeficient mouse models (F1) within three months post implantation, either in 16.9% (14/83) nude mice or in 26.9% (32/119) SCID mice, or from both species (3/5). Furthermore, 32 out of 49 F1 tumor tissues continued growth after implantation into nude mice (F2) and were passageable for more than three passages (≥F3), giving a final success rate of 15.5% (32/207).

To evaluate the potential impact of GC patient clinicopathological parameters on PDGCX model establishment success rate, we compared patients’ clinical parameters by dividing the GC patient tumors into ‘Established model’ and ‘Failed model’ groups as listed in Table 1. Statistical analysis revealed that the success rate of model establishment was independent of most patients’ pathological parameters such as age, tumor grade, clinical stage, TNM status, recurrence status and overall survival. However, mouse models were more likely to be successfully established if derived from a male patient’s GC tissue (*p* = 0.016) or from an intestinal GC (*p* = 0.030)

**Table 1:** Clinical characteristics of primary gastric carcinomas.

Parameters	Evaluable patient number	Unevaluable patient number*		Established model	Failed Model	*p*-value
Gender	161	46				0.016^a^
			Male	26	83	
			Female	4	48	
Median Age	195	12		62	62	0.532^b^
Tumor grade	158	49				0.271^a^
			2	12	33	
			3	16	87	
			4	2	8	
Clinical stage	160	47				0.587^a^
			1	0	4	
			2	10	29	
			3	14	70	
			4	6	27	
Tumor Size	161	46				0.729^a^
			T1	0	2	
			T2	2	7	
			T3	28	115	
			T4	0	7	
Lymph node metastases	158	49				0.635^a^
			N0	9	27	
			N1	12	52	
			N2	4	28	
			N3	5	23	
Distant Metastases	161	46				0.643^a^
			M0	28	125	
			M1	2	6	
Lauren subtype	191	16				0.030^a^
			Intestinal type	16	41	
			Diffused type	11	86	
			Mixed type	5	32	
Site	158	49				0.415^a^
			Pylorus	15	67	
			Cardia	3	16	
			Body	9	43	
			Pylorus and body	0	4	
			Lesser curvature	1	0	
Recurrence	160	47				1.000^a^
			No recurrence	28	124	
			With recurrence	1	7	
Mean Overall Survival (Month)	138	69				0.347^c^
				14.0869	21	
			Std Error	1.31631	12.01009777	

^a^: Fisher exact test

^b^: Mann Whitney U Test

^c^: Log Rank Test

* patients had missing information

### Histological assessment

**Fig 1:**
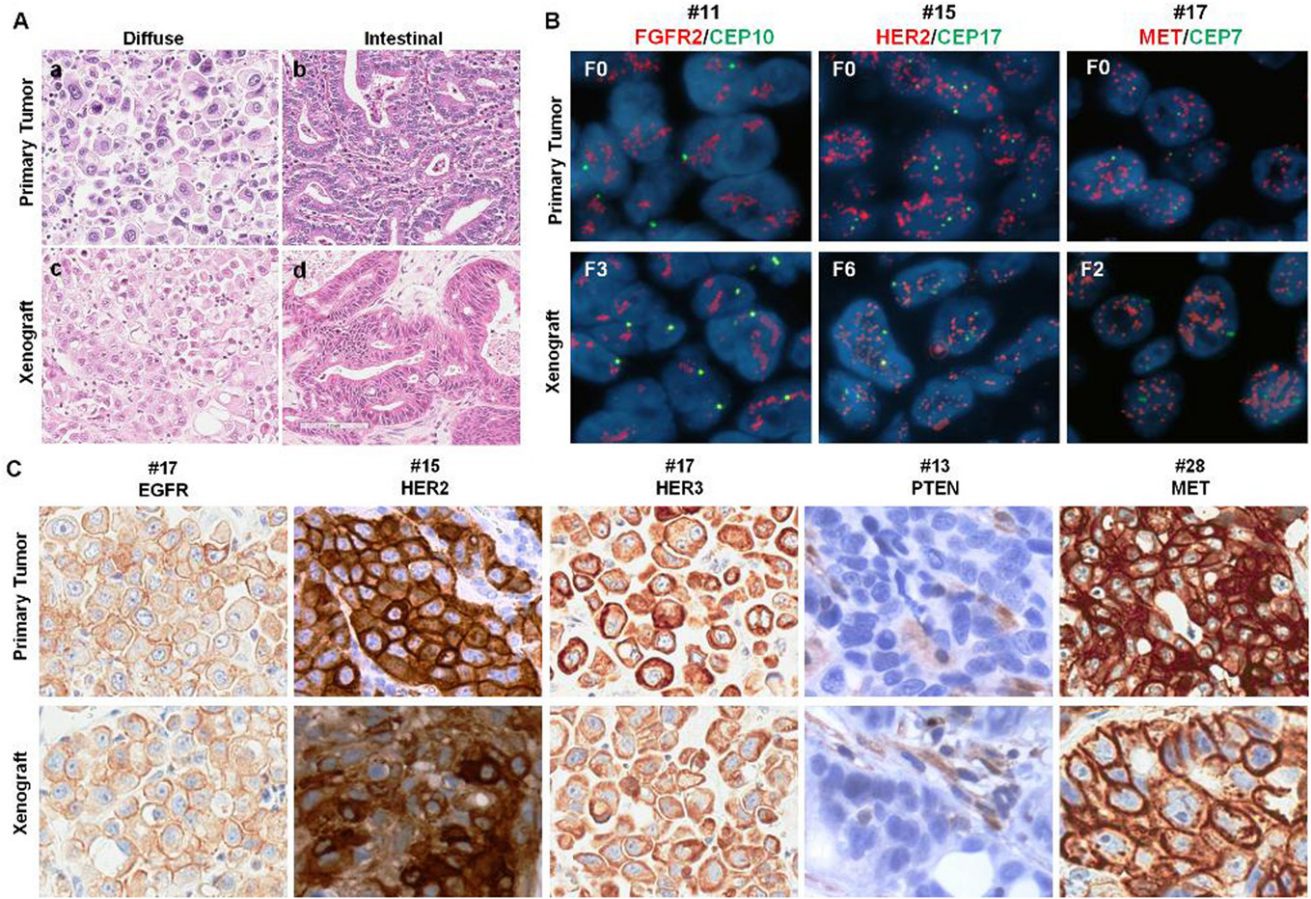
PDGCX models retain the histological features and genetic profiles of their parental tumors. (A) shows comparison of histological subtypes of GC observed in primary tumors (a, b) and corresponding PDGCX models (c, d); (B) shows *FGFR2*, *ERBB2* and *MET* gene amplification in primary tumors (F0) and corresponding PDGCX models. All target gene probes are labeled in red and CEP control probes are in green, nuclei are counterstained in blue by DAPI. (C) shows ERBB1 (+++), ERBB2 (+++), ERBB3 (+++), PTEN (-ve) and MET (+++) expression on primary tumors and corresponding PDGCX models.

Histological H&E assessment was performed by pathologist for all 207 GC patient tumors and 32 established PDGCX models. 16 models were classified as intestinal subtype, 11 were diffuse subtype and 5 were mixed subtype. Compared to the corresponding human tumors, all 32 PDGCX models perfectly maintained the same histological features as their parental human tumors (Fig 1A).

### Genetic characterization

To explore the genetic characteristics of the established PDGCX models, we focused on analysis of well-characterized driver oncogenes in GC including: the ERBB family members (*ERBB1*, *ERBB2* & *ERBB3*), *PTEN*, *FGFR2* and *MET* as listed in S1 Table. Among the 32 PDGCX models, 78% (25/32) of models showed positivity for ERBB1, 22% (7/32) were positive for ERBB2, 78% (25/32) were ERBB3 positive, 66% (21/32) lost PTEN expression, 3% (1/32) harbored *FGFR2* amplification, 41% (13/32) were MET expression positive, and 16% (5/32) were defined as *MET* amplified.

The genetic profile concordance between the PDGCX models and their parental patient tissues was analyzed either by agreement rate according to biomarker positivity, or by statistical analysis using the Cohen’s kappa coefficient. Between the PDGCX models and their corresponding primary tumors, a high degree of concordance was observed at the DNA level for *FGFR2* and *MET* genes (agreement rate = 100%; kappa value = 1) (Fig 1B); *ERBB2* status (combining FISH and IHC data together) showed almost perfect agreement (agreement rate = 94%; kappa value = 0.82) (Fig 1B and 1C); MET expression positivity and PTEN loss defined by IHC showed moderate agreement (agreement rate = 78%; kappa value = 0.46~0.56), whilst ERBB1 and ERBB3 expression showed only slight agreement (agreement rate = 59~75%; kappa value = 0.18~0.19) by IHC (Fig 1C) (S1 Table).

### PDGCX model genetic stability

**Fig 2:**
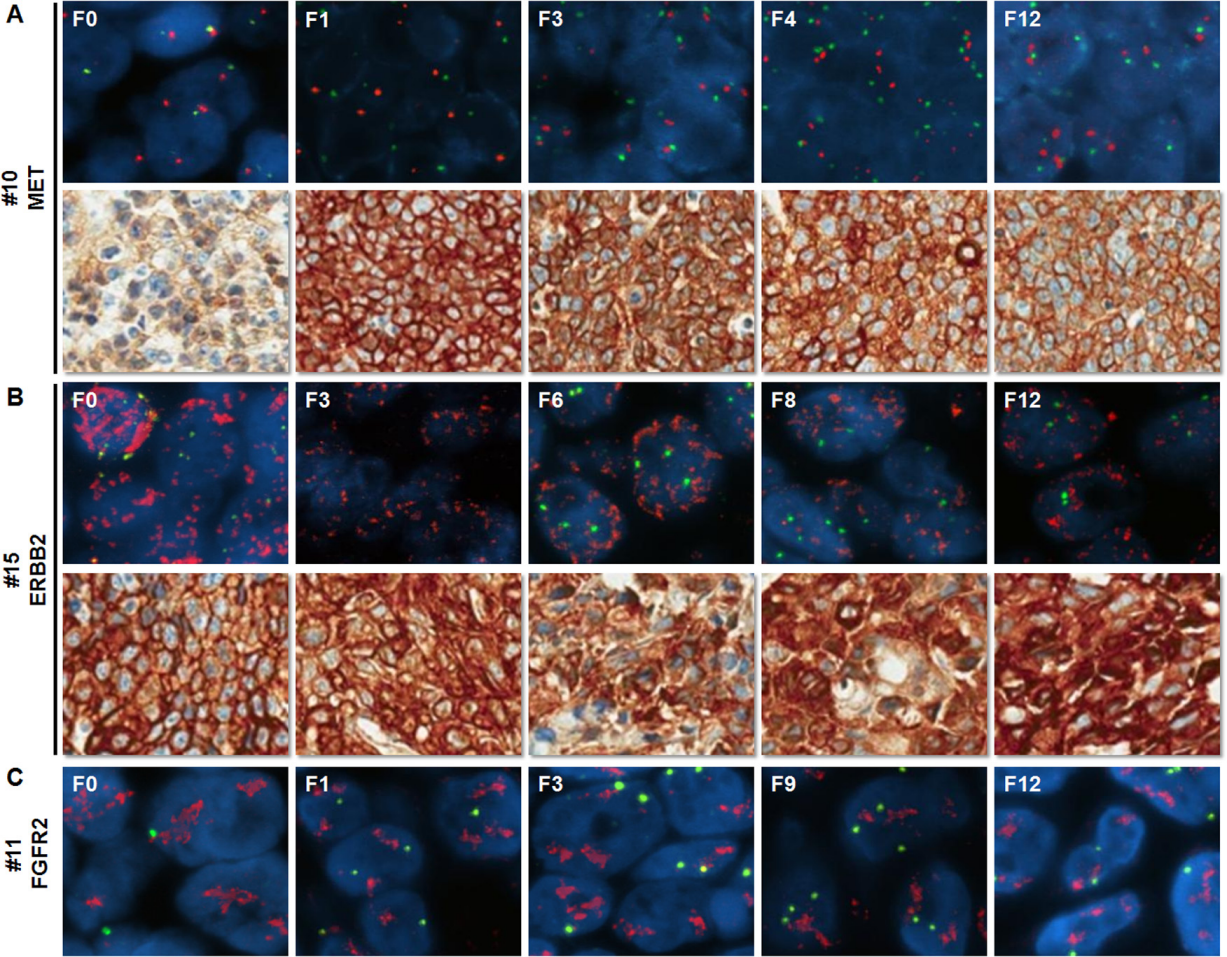
Biomarker profiles of serial passages of PDGCX models. Representative images of *MET* status (A), *ERBB2* status (B) and *FGFR2* gene amplification (C) on serial passages of PDGCX models by FISH or IHC are shown. All target gene probes for FISH are labeled in red and CEP control probes are in green, nuclei are counterstained in blue by DAPI.

To evaluate the genetic stability of the various biomarkers through sequential passages of established PDGCX models, we examined three biomarkers from different passages of 5 biomarker positive models. ERBB2 and MET protein expression, as well as *FGFR2*, *MET* and *ERBB2* gene amplification were continuously maintained in all generations of the same models up until the 12^th^ passage (Fig 2).

## Discussion

PDCX models from a variety of cancer types have been successfully established in recent years. We previously reported successful establishment of a large cohort of PDCX models from non-small cell lung cancer (NSCLC) and esopheageal cancers (EC) [17, 25]. However, the success rates for PDCX model establishment from different tumor types vary considerably, ranging from 5% to 80% [44]. For gastric cancer, only a few successful PDGCX models have been reported, indicating the difficulty of PDGCX establishment using human surgical tumor samples [33–36]. In our present study, we successfully established 32 PDGCX models, passagable to at least F3. To improve this success rate, we implanted human GC tissues simultaneously into nude and SCID mice at F0. Comparison of this data showed a trend towards a higher success rate when using SCID mice, compared to the use of nude mice either for the F1 generation (28.2% vs. 19.3%) or for the final successful establishment (16.9% vs. 13.6%), although these differences were not statistically significant. This data suggests that the more severe immunodeficient species may offer a superior platform for successfully establishing a potential PDGCX model. In the current study, we also observed that GC tissues from male patients (*p* = 0.016) or of intestinal subtype (*p* = 0.030) were easier to grow up in mice (Table 1). Interestingly, gender and Lauren subtype were highly correlated (*p* = 0.02589) in this cohort. The majority of the female patients (40 out of 49) presented with diffuse tumors, while the majority of patients with intestinal subtype tumors were male (38 out 47). Therefore, it is difficult to judge which factor(s) are dominant in determining the primary tumor’s potential to grow in mice.

The biomarkers studied here were selected based on existing known targets or potential new targets for clinical therapeutic strategies in GC. As molecular targets for guided therapeutics, these biomarkers are largely oncogenic drivers in GC, driven by either genomic or protein expression aberrations (or both). Therefore, the consistency of expression of these markers between human GC and PDGCX models, and among different generations within PDGCX models, is of importance in evaluating the utility of targeted therapeutic drugs. To explore how well this panel of 32 PDGCX models represented human GC, we performed further genetic characterization studies using IHC and FISH assays, in order to provide an *in situ* analysis of the target gene’s copy number and protein expression. The individual PDGCX model’s histological and genetic profiles were compared with the parental human GC tumors and agreement rates determined according to biomarker positivity and by Cohen’s Kappa. Our data revealed either high or perfect agreement in the majority of the biomarkers tested by either method, especially at the DNA level. Although ERBB1 and ERBB3 protein expression were judged to have ‘slight agreement’ according to kappa value, these biomarkers still showed an agreement rate of 59% and 75% between parental tumors and PDGCX models. A closer look at the data revealed that parental tumors and PDGCX models agreed well for both protein biomarkers, when the parental tumor was positive for the biomarker (14/16 for ERBB1 and 22/27 for ERBB3). This link was less obvious when the primary tumor was negative for the biomarker (5/16 for ERBB1 and 2/5 for ERBB3). Thus, the disagreement judged by Cohen’s Kappa could be a consequence of statistical bias due to the relatively small number of primary tumor negative samples. Furthermore, intratumoral heterogeneity of different biomarkers has been frequently reported in surgical GC samples [45–49], and may represent a more intrinsical reason for the inconsistencies between primary tumors and models. Nevertheless, the profiles of all tested biomarkers across the whole panel of 32 PDGCX models accurately reflects that of their prevalence in human GC samples, which is reported as 30~75% for ERBB1 positivity [50, 51], 15~22% for ERBB2 positivity [37, 50, 51], 60~70% for ERBB3 high expression [51, 52], 39~47% for PTEN loss [53, 54], 4–5% for *FGFR2* amplification [8, 9, 37, 55], 20~70% for MET high expression [37, 56–59] and 0~10% for *MET* amplification [37, 56, 58–60]. This high concordance could in part be attributable to the lack of correlation between most of the patient clinicopathological parameters and model success rates (i.e. There appears to be little bias on model success, so most patient tumors should give rise to viable PDGCX models). Importantly, the positivity of ERBB2, FGFR2 and MET, either at the DNA or protein level, were stably maintained in all PDGCX models, out to at least passage F12. This considerable fidelity and duration of genetic maintenance underscores another advantage of using PDGCX models for evaluation of pre-clinical drug efficacy.

The purpose of generating this panel of PDGCX models was to evaluate the anti-tumor efficacy of targeted therapeutic drugs. Indeed, a number of PDGCX models from this panel with different genetic aberrations have been previously used to successfully explore preclinical drug efficacy [9, 37–40]. For example, models with positive ERBB2 (#8 and 16), *FGFR2* amplification (# 11), PTEN loss (#2) and *MET* amplification (#10, 18 and 30) were sensitive to Trastuzumab, AZD4547, AZD5363 and Volitinib, respectively. Taken together, these data highlight the considerable utility of PDGCX models in evaluating preclinical drug efficacy, enabling definition of prospective biomarker selection criteria and modeling of tumor architecture and genetic heterogeneity.

## Conclusions

In summary, we have successfully established a panel of 32 PDGCX models and demonstrated that these models faithfully recapitulate the histological characteristics and genetic diversity of the primary human tumors. Furthermore, these PDGCX xenograft models maintain genetic diversity until at least the 12th model passage. Our data also show that the panel in its entirety accurately reflects the tested biomarker profiles present in the wider human GC population. Thus, this panel of PDGCX models represents a valuable tool in understanding this lethal disease and serves as a powerful resource in enabling preclinical efficacy testing in oncology drug discovery.

## Supporting Information

S1 Table Molecular characteristics of PDGCX and the corresponding parental tumors. (DOC)
