# Supplementary material for: Patient-Derived Gastric Carcinoma Xenograft Mouse Models Faithfully Represent Human Tumor Molecular Diversity
Source: PLoS One. 2015 Jul 28;10(7):e0134493. doi: 10.1371/journal.pone.0134493 (PMC4517891; doi:10.1371/journal.pone.0134493)
Supplement: S1 Table — (DOC) [file pone.0134493.s001.doc]

**Supporting Table 1. Molecular characteristics of established xenografts and parental tumors.**

| **No.** | **ERBB1 (IHC)** | | **ERBB2 (IHC + FISH)** | | **ERBB3 (IHC)** | | **PTEN loss (IHC)** | | **FGFR2 (FISH)** | | **cMET (IHC)** | | **cMET (FISH)** | |
| --- | --- | --- | --- | --- | --- | --- | --- | --- | --- | --- | --- | --- | --- | --- |
|  | **Primary** | **Xenograft** | **Primary** | **Xenograft** | **Primary** | **Xenograft** | **Primary** | **Xenograft** | **Primary** | **Xenograft** | **Primary** | **Xenograft** | **Primary** | **Xenograft** |
| 1 | 0 | 2 | -ve | -ve | 1 | 2 | 2 | 2 | -ve | -ve | 0 | 0 | -ve | -ve |
| 2 | 1 | 3 | -ve | -ve | 0 | 2 | 0 | 0 | -ve | -ve | 0 | 0 | -ve | -ve |
| 3 | 3 | 3 | -ve | -ve | 0 | 2 | 0 | 0 | -ve | -ve | 0 | 0 | -ve | -ve |
| 4 | 0 | 3 | -ve | -ve | 2 | 3 | 1 | 1 | -ve | -ve | 0 | 0 | -ve | -ve |
| 5 | 0 | 2 | -ve | -ve | 3 | 2 | 2 | 1 | -ve | -ve | 3 | 3 | +ve | +ve |
| 6 | 2 | 2 | -ve | -ve | 3 | 2 | 0 | 2 | -ve | -ve | 0 | 0 | -ve | -ve |
| 7 | 3 | 2 | -ve | -ve | 2 | 3 | 0 | 0 | -ve | -ve | 0 | 0 | -ve | -ve |
| 8 | 0 | 3 | -ve | +ve | 3 | 3 | 0 | 0 | -ve | -ve | 1 | 1 | -ve | -ve |
| 9 | 1 | 2 | -ve | -ve | 0 | 0 | 2 | 0 | -ve | -ve | 2 | 3 | -ve | -ve |
| 10 | 1 | 3 | -ve | -ve | 0 | 0 | 1 | 0 | -ve | -ve | 3 | 3 | +ve | +ve |
| 11 | 3 | 2 | -ve | -ve | 2 | 2 | 2 | 1 | +ve | +ve | 2 | 0 | -ve | -ve |
| 12 | 3 | 3 | -ve | -ve | 3 | 2 | 1 | 2 | -ve | -ve | 2 | 3 | -ve | -ve |
| 13 | 0 | 3 | -ve | -ve | 3 | 1 | 0 | 0 | -ve | -ve | 2 | 1 | -ve | -ve |
| 14 | 3 | 3 | -ve | -ve | 3 | 2 | 0 | 0 | -ve | -ve | 2 | 1 | -ve | -ve |
| 15 | 0 | 2 | +ve | +ve | 2 | 3 | 1 | 1 | -ve | -ve | 0 | 0 | -ve | -ve |
| 16 | 2 | 3 | +ve | +ve | 3 | 1 | 2 | 1 | -ve | -ve | 3 | 1 | -ve | -ve |
| 17 | 0 | 3 | -ve | -ve | 3 | 3 | 1 | 1 | -ve | -ve | 0 | 0 | -ve | -ve |
| 18 | 3 | 3 | -ve | -ve | 3 | 3 | 3 | 3 | -ve | -ve | 3 | 3 | +ve | +ve |
| 19 | 3 | 3 | -ve | -ve | 3 | 3 | 3 | 3 | -ve | -ve | 3 | 3 | +ve | +ve |
| 20 | 3 | 3 | -ve | -ve | 3 | 3 | 0 | 0 | -ve | -ve | 0 | 0 | -ve | -ve |
| 21 | 2 | 3 | +ve | -ve | 3 | 3 | 0 | 0 | -ve | -ve | 0 | 0 | -ve | -ve |
| 22 | 3 | 3 | +ve | +ve | 3 | 3 | 2 | 2 | -ve | -ve | 3 | 3 | -ve | -ve |
| 23 | 2 | 1 | -ve | -ve | 2 | 2 | 0 | 0 | -ve | -ve | 0 | 0 | -ve | -ve |
| 24 | 0 | 2 | +ve | +ve | 3 | 3 | 0 | 0 | -ve | -ve | 2 | 0 | -ve | -ve |
| 25 | 2 | 1 | -ve | -ve | 3 | 2 | 1 | 0 | -ve | -ve | 1 | 2 | -ve | -ve |
| 26 | 1 | 1 | -ve | -ve | 3 | 1 | 0 | 0 | -ve | -ve | 3 | 2 | -ve | -ve |
| 27 | 1 | 1 | -ve | -ve | 3 | 3 | 1 | 0 | -ve | -ve | 0 | 0 | -ve | -ve |
| 28 | 1 | 1 | -ve | -ve | 3 | 1 | 0 | 0 | -ve | -ve | 1 | 3 | +ve | +ve |
| 29 | 2 | 2 | -ve | -ve | 2 | 2 | 1 | 0 | -ve | -ve | 2 | 2 | -ve | -ve |
| 30 | 2 | 2 | -ve | -ve | 3 | 2 | 2 | 0 | -ve | -ve | 3 | 3 | +ve | +ve |
| 31 | 2 | 2 | +ve | +ve | 3 | 2 | 1 | 0 | -ve | -ve | 2 | 2 | -ve | -ve |
| 32 | 1 | 1 | +ve | +ve | 3 | 3 | 1 | 0 | -ve | -ve | 2 | 2 | -ve | -ve |
| **Positive rate** | **50%** | **78%** | **22%** | **22%** | **84%** | **78%** | **44%** | **66%** | **3%** | **3%** | **50%** | **41%** | **16%** | **16%** |
| **Agreement rate** | 59% (19/32) | | 94% (30/32) | | 75% (24/32) | | 78% (25/32) | | 100% (32/32) | | 78% (25/32) | | 100% (32/32) | |
| **Kappa value** | 0.19 | | 0.82 | | 0.18 | | 0.46 | | 1.00 | | 0.56 | | 1.00 | |
